# The Clausius–Mossotti Factor in Dielectrophoresis: A Critical Appraisal of Its Proposed Role as an ‘Electrophysiology Rosetta Stone’

**DOI:** 10.3390/mi17010096

**Published:** 2026-01-11

**Authors:** Ronald Pethig

**Affiliations:** Institute for Integrated Micro and Nano Systems, School of Engineering & Electronics, The University of Edinburgh, The King’s Buildings, Edinburgh EH9 3JF, UK; ron.pethig@ed.ac.uk

**Keywords:** Clausius–Mossotti factor, dielectrophoresis, Laplace’s equation, polarization and local field theory

## Abstract

The Clausius–Mossotti (CM) factor underpins the theoretical description of dielectrophoresis (DEP) and is widely used in micro- and nano-scale systems for frequency-dependent particle and cell manipulation. It has further been proposed as an “electrophysiology Rosetta Stone” capable of linking DEP spectra to intrinsic cellular electrical properties. In this paper, the mathematical foundations and interpretive limits of this proposal are critically examined. By analyzing contrast factors derived from Laplace’s equation across multiple physical domains, it is shown that the CM functional form is a universal consequence of geometry, material contrast, and boundary conditions in linear Laplacian fields, rather than a feature unique to biological systems. Key modelling assumptions relevant to DEP are reassessed. Deviations from spherical symmetry lead naturally to tensorial contrast factors through geometry-dependent depolarisation coefficients. Complex, frequency-dependent CM factors and associated relaxation times are shown to inevitably arise from the coexistence of dissipative and storage mechanisms under time-varying forcing, independent of particle composition. Membrane surface charge influences DEP response through modified interfacial boundary conditions and effective transport parameters, rather than by introducing an independent driving mechanism. These results indicate that DEP spectra primarily reflect boundary-controlled field–particle coupling. From an inverse-problem perspective, this places fundamental constraints on parameter identifiability in DEP-based characterization. The CM factor remains a powerful and general modelling tool for micromachines and microfluidic systems, but its interpretive scope must be understood within the limits imposed by Laplacian field theory.

## 1. Introduction

This article appraises the proposal by Hughes [1] that the Clausius–Mossotti (CM) Factor can serve as an Electrophysiology Rosetta Stone. The physical object is a stele discovered in Egypt at el-Rashid (Rosetta). Carved on it is a triplication of a decree of loyalty to King Ptolemy written in hieroglyphs and demotic and ancient Greek, and was the key to deciphering ancient Egyptian scripts. Hughes proposes that the CM factor can serve to unify, within one cellular electrome, the three biophysical methods of dielectrophoresis (DEP), patch clamp determination of membrane potential, and zeta potential measurements.

In DEP studies the complex CM factor, K*(ω), is given for a spherical particle as

(1) $${\left[CM\;Factor\right]}_{DEP}\equiv K^{*}\left(\omega \right)=\frac{\varepsilon_{p}^{*}-\varepsilon_{m}^{*}}{\varepsilon_{p}^{*}+2\varepsilon_{m}^{*}}$$

It is derived by solving Laplace’s equation, with specific boundary conditions, for the electric potential around a polarized dielectric sphere of complex permittivity, $\varepsilon_{p}^{*}$, located within an external medium of complex permittivity $\varepsilon_{m}^{*}$. The DEP force equation employs the real component (1.0 > Re K(ω) > −0.5) because it uses the scalar (dot) product, (p·∇)E, to describe the interaction between the induced dipole moment, p, and the gradient of the non-uniform field E. The imaginary components cancel out. Hughes argues, on the basis of experimental support, e.g., [2,3], that by analyzing the DEP behaviour of a cell suspension for different settings of the field frequency, ω, and conductivity of the suspending medium, a determination of K(ω) can not only provide the passive electrical properties (membrane capacitance, membrane conductance, cytoplasm conductivity), but also the membrane potential (Vm) and zeta potential (ζ). It is suggested that this has implications for the redesign of DEP cell separators—especially for operation at high–medium conductivities and separation protocols based on the strong negative DEP response of either the target or unwanted cells [1].

The electrome, broadly referring to the bioelectrical and electromagnetic signalling system of an organism, has gained traction for plant electrophysiology [4]. If applied to mammalian cells, this could lead to deeper insights of how they regulate not just their internal ionic state, but also their surface electrostatic environment. These are important aspects of cell adhesion and signalling, immunology, and metastasis, for example. The capability to monitor, with high throughput, Vm or ζ with DEP would be powerful in some clinical and biotechnological applications. However, as yet, Hughes’ potentially powerful “Rosetta Stone” metaphor has not been derived from first principles, and relies on several assumptions and empirical fits. Broader validation and mechanistic groundings are required. For example, does the framework for the model apply solely to a static or quasi-static membrane potential? To what extent could dynamic changes in Vm occurring in depolarization and repolarization, as measured by patch-clamp or potentiometric dyes [5], be mapped onto changes in K(ω) measured in real-time? The proposal to use K(ω) of Equation (1) as a unifying “Rosetta Stone” is centred on whether it can meaningfully bridge fundamentally different biophysical and physiological measurements. In this respect, the author is mindful of Faraday’ remark [6]:

*‘Who can forget that Mossotti has shown that gravitation, aggregation, electric force and electrochemical action may all have one common connexion or origin, and so, in their actions at a distance, may have in common that infinite scope which some of their actions are known to possess?’*

**Figure 1:**
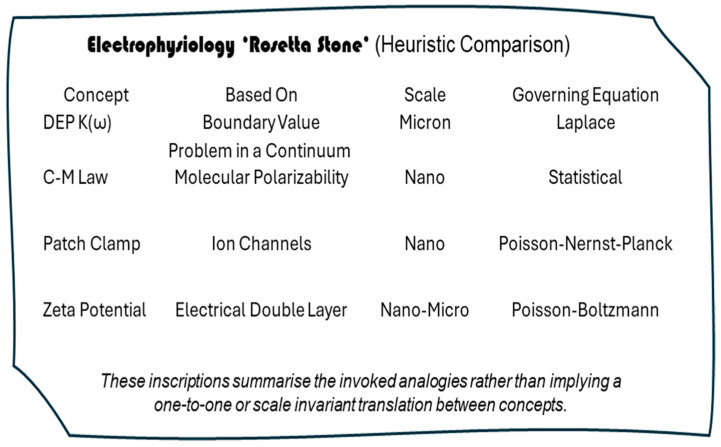
The ‘Electrophysiology Rosetta Stone’ metaphor, depicted as inscriptions on a stele giving the implied analogies between DEP ‘contrast factors’ and electrophysiological descriptors across scales. As discussed in this paper, these correspondences provide heuristic unification through shared electrostatic principles, but do not imply a literal or scale-invariant translation between field-mediated response and intrinsic biological processes.

It has been said that *‘Maths relies on physics to give it useful things to do’*. ([7], p. 203). Faraday reminds us that the math can work even when the physics is erroneous. The situation can also arise where ‘*Either we don’t have the right math to merge the two theories, or the way we are merging them is wrong.’* ([7], p. 297). An appraisal of the “Rosetta Stone” metaphor faces the risk of being misleading if limitations of the metaphor are not clearly understood by bioengineers and biophysicists, and are not clearly explained to electrophysiologists and cell biologists. The author is no Jean-François Champollion, but Figure 1 is offered as an initial starting point for this exercise.

A key point to unravel and appreciate is that, as indicated in Figure 1, two different factors associated with two very different physical contexts carry the Clausius–Mossotti label. K(ω) of Equation (1) is a mathematical contrast factor that describes the extent to which the induced dipole moment in a sphere strengthens or weakens the external electric field. The physics involved deals solely with bulk material properties (complex permittivity), with no consideration given to molecular structure. K(ω) was repurposed for DEP, even though, as discussed here, the math and physics associated with it differ significantly from those applied by Mossotti [8,9] and Clausius [10] to connect the dielectric constant of a material to the number density of constituent molecules and their molecular polarizability. The physics for this involves statistical mechanics, electromagnetism, and that of a microscopic dipole interacting with a self-consistent local electric field—with bulk material being treated as an assembly of polarizable dipoles. Historically (from the author’s perspective) the tension between these two CM’s appears to have arisen after Arnold et al. [11] introduced the use of K(ω) in electrorotation theory, where they refer to it as ‘effectively a macroscopic application of the Clausius–Mossotti factor’. The imaginary part of K(ω) is relevant to electrorotation because the torque acting on a particle is the vector cross product of the induced moment and rotating field. Jones [12] then adopted this CM label to replace the term ‘relative dielectrophoresis force factor’ used by Pohl in his seminal text ([13], p. 36).

For clarity, in this article, a distinction will be made between the Molecular Clausius–Mossotti Relation and the DEP Clausius–Mossotti Factor. Notation that employs an asterisk, as in ε*, signifies a complex physical quantity having both real and imaginary components. Absence of the asterisk, as in ε, σ, for example, indicates a real quantity with no imaginary, component.

## 2. The Molecular Clausius–Mossotti Relation

As described in a selection of relevant textbooks, the following expression

(2) $$\frac{\varepsilon -1}{\varepsilon +2}=C\rho$$

is variously referred to as the Clausius–Mossotti Law [14], Clausius–Mossotti Equation [15,16,17,18,19,20,21], Clausius–Mossotti Relation [12,22,23], or the Clausius–Mossotti Formula [24,25,26,27]. As explained in these texts, Equation (2) provides an acceptable description of the dependence of the dielectric constant (relative permittivity) *ε* on density *ρ* for *non-polar* gases, liquids, and some solid dielectrics. The constant *C* is given by

(3) $$C=\frac{N_{A}\alpha }{3M}$$

where *M* is the molecular weight of the material, *N*_A_ is Avogadro’s number, and α is the molecular polarizability, defined by the equation

(4) $$p=\alpha \varepsilon_{o}E_{eff}$$

where **p** is the dipole moment induced in a single molecule, *ε*_o_ is the permittivity of free space, and **E***_eff_* is the effective local electric field force acting on a single molecule inside the bulk material. The math and physics have evolved to become ‘effective medium’ theories [28], having widespread applications for composite materials and condensed matter in general [29,30,31,32,33,34,35]. Choy [36] has shown that the hydrodynamic equivalent to the Clausius–Mossotti formula provides new theories for the rheology of sol-gels, polymeric solutions, and magnetic fluids, whilst Almog [37] provides rigorous proof of the formula and an application of the effective medium theory for steady heat flow.

### 2.1. The Contribution of Ottaviano Fabrizio Mossotti

Mossotti’s 1836 paper [8] was strongly influenced by the ideas of Franklin [38] and Cavendish [39], and this is summarized in a letter written to Friedrich Zöllner [40] on 22 April 1880, by Wilhelm Weber (who had shown the velocity of light to be equal to the ratio of the electromagnetic and electrostatic units of charge [41]). The translation of this letter reads as follows:

*‘Dear Friend. I thank you very much for the kind communication of Mossotti’s paper, which I was very eager to become acquainted with, although I did not find in it what I expected—namely, the idea first communicated to me by you, that ponderable molecules can be regarded as compounds of positive and negative electrical particles, with the additional assumption that the attractive force between unlike particles is somewhat greater than the repulsive force between like particles, so that the law of gravitation follows from the fundamental electrical law. This idea is not contained in Mossotti’s paper, and Mossotti could not arrive at this idea, because he is completely devoted to the Franklin–Aepinus view, according to which not two but only one kind of electricity exists, which he regards as an aether filling space continuously, whose atoms mutually repel one another. Instead of ponderable bodies he assumes only isolated material molecules, surrounded on all sides by aether, and he attributes to these molecules mutual repulsion, and to their interaction with the aether atoms attraction. From the combined action of these forces he derives for the material molecules, at large distances, the law of gravitation, and at a certain very small distance, on the other hand, a stable equilibrium position, the demonstration of which he regards as the foundation of his theory of the molecular constitution of bodies, to which he attaches the greatest importance.’*

In this letter Weber refers to Franz Aepinus, a mathematician whose ideas were based on Benjamin Franklin’s one fluid theory, but with a Newtonian action-at-a-distance replacing Franklin’s electrical atmospheres [42].

For his 1850 paper [9], Mossotti retained his ideas of action at a distance of aethereal electricity, and employed the earlier mathematical formalism developed by Poisson [43] for magnetism, to the extent that Maxwell states the following [44]:

*‘Thus, when Mossotti observed that certain quantities relating to electrostatic induction in dielectrics had been shewn by Faraday to be analogous to certain quantities relating to magnetic induction in iron and other bodies, he was enabled to make use of the mathematical investigation by Poisson relative to magnetic induction, merely translating it from magnetic language into the electric, and from French into Italian’.*

This remark of 1871 is ungracious, but perhaps understandably so. By 1865, Maxwell had developed his revolutionary theory of electromagnetism [45]. The “action-at-a-distance” models of Poisson and Mossotti, with their fluids and aethers were outdated. Dismissing the work as just a translation from French into Italian may factually be correct, but overlooks that Mossotti openly states ([9], p. 51) that, for his theory, “*we can use that which the famous Poisson provided—when treating the theory of magnetism”*. He explicitly draws an analogy between Poisson’s theory of magnetism and the new theory of dielectrics. He models each ‘corpuscle’ of the dielectric as having a polarizable “atmosphere” of aether (or electric fluid). If *ρ* is the distance from a volume element dψdξdς in the dielectric to the electricity source, then $\mu (d\psi d\xi d\varsigma )/\rho^{2}$ is the force exerted on the electricity by dψdξdς. The polarity of the induced corpuscular dipole moment is determined by the factor *μ*, which is positive if the density of dψdξdς is greater than the equilibrium value, and negative if less. Following Poisson, he then employs a sophisticated averaging process to calculate the net effect of all these induced dipoles throughout the entire dielectric volume. From this, Mossotti reaches his most important conclusions ([9], pp. 67–74, Equations (14)–(18)): (i) the macroscopic effect of the entire polarized dielectric is exactly equivalent to a fictitious distribution of charges on the surfaces of the conductors immersed within it; (ii) the sum of all these fictional charges is zero; (iii) these surface charge layers act on the conductors as if they were isolated and acting at a distance, without the dielectric; (iv) the total free charge (on the conductors plus those induced on the walls of the dielectric) is conserved.

Thus, Mossotti did not just copy Poisson’s physics of a two-fluid magnetic model, but adopted it for his own model of a single-fluid atmosphere, making the conceptual leap in realizing that the same mathematical structure could be applied to the completely different physical phenomenon of electric dipoles in dielectrics. He was the first to realize this. His innovation was not in solving the same differential equations as Poisson, but in recognizing that they were the right ones to solve. For example, he derives (but does not solve) a differential equation for a new potential function *ϕ* ([9], p. 66, Equation (10)):

(5) $$\frac{d^{2}\phi }{dx^{2}}+\frac{d^{2}\phi }{dy^{2}}+\frac{d^{2}\phi }{dz^{2}}=0$$

For a homogeneous dielectric, Equation (5) is Laplace’s equation ($\nabla^{2}V=0$). He also introduced the concept of a cavity field *inside* the dielectric. For this, he conceived of a small sphere inside the dielectric. The total electric field inside this cavity is the sum of the field from all charges *outside* the cavity plus the field from the dipoles *inside* the cavity. He calculated the contribution from the dipoles on the cavity’s surface and found it to be 4π/3)*k*(α, β, γ) ([9], p. 61, Equation (4)), where the continuous polarization vector field within the dielectric is given by (*kα*, *kβ*, *kγ*) and *k* is the ratio of electrical fluid (aether) volume to the total volume of the dielectric. Von Hippel refers to this as the ‘Mossotti field’ ([18], pp. 98 and 176) and is the framework that Clausius later refined [10].

### 2.2. The Contributions of Rudolf Clausius

Mossotti provided the mathematical bridge between Poisson’s theory of magnetism and the polarization of dielectrics. Clausius, in volume 2 (The Mechanical Treatment of Electricity) of his book *The Mechanical Theory of Heat* [10], reformed this using more modern assumptions concerning the equilibrium configuration of molecular structures, placing it on the firmer thermodynamic and electromagnetic footing now described in standard textbooks, e.g., [15,16,17,18,19,20,21,22,23,24,25,26]. He abandoned the explicit language of an all-pervading electrical aether and instead treated matter as an assembly of discrete, polarizable molecules embedded in an otherwise electrically uniform medium. A description of this, based on a German–English translation, is given elsewhere [46], and what follows is an embellished summary.

Clausius employs Green’s theorem connecting surface and volume integrals that incorporate the continuity of electric potential gradients across infinitesimally thin ‘mathematical surfaces’ [47], together with Faraday’s concept of specific inductive capacity [48] and Maxwell’s treatment of electricity in terms of elastically bound charges [49]. He assumes that each molecule, when subjected to an external electric field, acquires an induced dipole moment proportional to the local electric field at its position. The key difficulty, which Clausius confronted more rigorously than Mossotti, was in defining this effective, local field that acts on any given molecule. This is not just the applied field, but the vector sum of the applied field and the fields due to all neighbouring polarized molecules. To solve this difficulty, Clausius adopted a conceptual construction similar to (but more refined than) Mossotti’s cavity argument. He considered a small spherical region carved out of a uniformly polarized dielectric. Within this cavity, the field acting on a test molecule is equal to the macroscopic field plus a contribution arising from the surface polarization charges on the cavity boundary. For a spherical cavity, this additional field is (in modern notation)

(6) $$E_{local}=E_{lmacro}+\frac{P}{3\varepsilon_{o}}$$

where P is the polarization of the medium and ε_0_ is the permittivity of free space. This result, later known as the Lorentz local field ([50], pp. 137–138), provides the crucial link between the microscopic polarization of individual molecules and the macroscopic electric displacement of the bulk material.

Clausius derives (without solving Laplace’s equation) the following relationship:

(7) $$K=\frac{1+2g}{1-g}$$

where K is Faraday’s specific inductive capacity parameter [48]. Parameter g is the ratio v_c_/V_d_ of the total volume of all the spherical particles to that of the total dielectric, and is thus a function of the mass density *ρ* of the dielectric. It serves as a simplifying factor in relating the corpuscle’s induced polarization and the electric field in the surrounding dielectric medium ([10], p. 94). Clausius applies his formulae to measurements of the charge stored in Leyden jars and on the plates of Franklin’s tables, concluding that the surface area of a plate is more important than its radius of curvature. He determines the ratio of potential differences between plates with and without inserted polarized glass to be equivalent to K (i.e., K = ε). A simple manipulation of Equation (7) thus gives Equation (2). The dielectric constant of a material is thus shown as no longer simply a phenomenological parameter, but a quantity directly tied to molecular structure and mass density of the dielectric. Clausius therefore completed an important conceptual transition. Mossotti showed that a polarized dielectric could be treated, mathematically, as a continuous distribution of induced dipoles giving rise to surface charge densities and internal fields. Clausius, by contrast, demonstrated how these macroscopic quantities emerge from a discrete ensemble of polarizable entities. In doing so, he linked electrostatics with early molecular theory and provided a route by which dielectric measurements could, in principle, inform on molecular size, bonding, and structure.

### 2.3. Membrane Electrostriction

The electrostatic stress (force), **f**, acting on an element of a dielectric is [14,23]

(8) $$f=QE-\frac{\varepsilon_{o}}{2}E^{2}\nabla \varepsilon +\frac{\varepsilon_{o}}{2}\nabla \left(E^{2}\rho \frac{\partial \varepsilon }{\partial \rho }\right)$$

where *Q* is the net charge density carried by that element. The term *QE* produces an electrophoretic force. The aqueous medium used to suspend cells in DEP studies can be assumed to be incompressible. In response to physical stress, the volume of the medium therefore remains fixed, so that, in Equation (2), the density *ρ* of the media is constant. However, this is not true for cell lipid membranes: they exhibit elasticity (e.g., under osmotic stress or during exocytosis) [51,52,53,54]. The term ∂ε/∂ρ in Equation (8) is obtained by differentiating Equation (2) to give

(9) $$\frac{\partial \varepsilon }{\partial \rho }=\frac{{\left(\varepsilon +2\right)}^{2}}{3}C=\frac{\left(\varepsilon +2)(\varepsilon -1\right)}{3\rho }$$

The contribution to the field energy, *W*, stored in a cell membrane from just the membrane potential, *V_m_*, is

(10) $$W=\frac{1}{2}C_{mem}V_{m}^{2}\;$$

where *C_mem_* is the membrane capacitance given for a spherical cell of radius, *R*, membrane thickness, *d_mem_*, and relative permittivity, ε_mem_, by

(11) $$C_{mem}=\frac{4\pi R^{2}}{d_{mem}}\varepsilon_{o}\varepsilon_{mem}$$

The resting membrane potential, *V*_m_, is not an equilibrium potential. It relies on a constant expenditure of energy, manifested as a flux of ions across the membrane. A membrane is ~7 nm thick, so that a membrane potential, *V*_m_, of −70 mV corresponds to an electric field strength (*V_m_*/*d_mem_*) of around 10 MV/m directed outwards from the cytoplasm towards the extracellular medium.

In addition to *V*_m_, a potential difference *V_mem_* is also created across the cell membrane by the applied AC electric field, as given by Equation (12):

(12) $$V_{mem}(\omega ,\theta )=\frac{3}{2}\frac{RE\cos\theta }{\left(1+i\omega \tau \right)}$$

where *θ* is the polar angle with respect to the direction of the field, *E*, and *τ* is the relaxation time of the induced interfacial polarization of the cell [55,56]. For an intact cell membrane exhibiting a membrane potential, its passive electrical conductivity is so small that, to a good approximation,

(13) $$\tau \approx \frac{RC_{mem}}{2\sigma_{i}\sigma_{e}}\left(\sigma_{i}+2\sigma_{e}\right)$$

where *σ_i_* and *σ_e_* are the electrical conductivities of the cell interior (cytoplasm) and extracellular medium, respectively [57]. As indicated by Equation (12) *V_mem_* is a complex quantity composed of a real and imaginary component, the latter having either a positive or negative value (depending on whether the polarization leads or lags the applied field, respectively). The field *E_mem_* created across the membrane is given by |*V_mem_*|/*d_mem_*. From Equation (12),

(14) $$E_{mem}(\omega ,\theta )=\frac{3}{2}\frac{(R/d)E\cos\theta }{(1+\omega^{2}\tau^{2}{)}^{1/2}}$$

**Figure 2:**
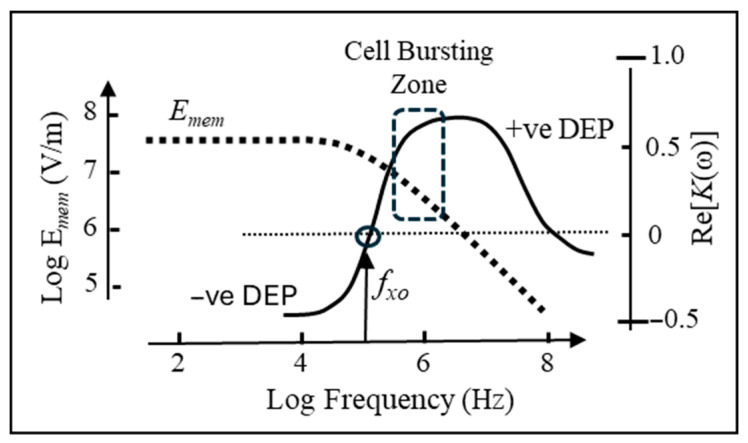
A plot of the transmembrane field *E_mem_*, derived from Equation (14) for an applied local field of 30 kV/m, is shown superimposed on a typical plot of the DEP response for lymphocytes in a medium of conductivity 40 mS/m [57]. The zone where cell bursting is commonly observed is indicated.

The function given by Equation (14) is plotted in Figure 2 and superimposed on the frequency variation in K(ω) given by Equation (1), as typically obtained for lymphocytes [56]. At low frequencies (*ωτ* < 1), the field *E_mem_* imposed across the membrane can exceed the applied field by a factor of 10^3^ or greater, depending on the ratio of the cell radius and membrane thickness. As the frequency is increased and approaches the DEP cross-over frequency, the magnitude of *E_mem_* starts to decrease. With increasing field frequency, the total voltage drop across the cell is increasingly shared by the cell interior and not just across the membrane. It is this frequency range (indicated in Figure 2) just beyond the DEP cross-over frequency, *f_xo_*, that it is common in DEP experiments to observe cells spinning at electrode edges under the influence of an induced rotational torque. Such behaviour can result in significant shear forces on the cell membrane, resulting in cell damage or bursting. Above a certain frequency, however, where the membrane field falls below 10 MV/m, the DEP force acting alone is normally not sufficient to initiate cell destruction [57,58]. If $f_{mf}=1/(2\pi \tau )$ is defined as the frequency where the absolute value of the induced membrane field decreases by a factor of 1/√2 from its maximum value given by Equation (14), the following relationship between *f_xo_* and *f_mf_* can be derived [57]:

(15) $$f_{xo}=\frac{f_{mf}}{\sqrt{2}}\left(1+\frac{2\sigma_{e}}{\sigma_{i}}\right)$$

This indicates that the close relationships between the DEP cross-over frequency and the fall in membrane field shown in Figure 2 can be expected to hold for the range (1–200 mS/m) of medium conductivities commonly employed in cell DEP experiments. This fact has been incorporated into the design and experimental protocols of commercial instruments, where the target cells (circulating tumour cells in peripheral blood) are attracted by positive DEP to a sample collection port integrated with an electrode array, e.g., [59].

If sufficiently strong, the electrostriction force could possibly (i) alter the conformation, and hence the pumping action, of the transmembrane voltage-gated ion channels and change *V_m_*, and (ii) alter the value of K(ω) through changes in the cytoplasm conductivity and permittivity resulting from induced leakage of ions from the cytoplasm into the extracellular medium. Such events would confound the interpretation of measurements designed to validate Hughes’ proposed electrome model. If the high fields and field gradients used to measure K(ω) can themselves alter *Vm* or the membrane integrity, this challenges the model’s validity for real-time, non-invasive translation.

A summary of the discussion so far is given in Table 1.

## 3. The DEP Clausius–Mossotti Factor (K(ω))

### 3.1. Spherical Surface Harmonic Functions

The factor K(ω), defined in Equation (1), is obtained by solving Equation (5) (i.e., Laplace’s Equation $\nabla^{2}\phi =0$), with appropriate boundary conditions, to determine the electrical potential *ϕ* at every point inside and outside the sphere. The general theory for solving Laplace’s equation is known as potential theory [60,61] and, as implied by Equation (5), solutions for it are twice (as well as continuously) differentiable. They are called surface spherical harmonics [49]. Equation (5) employs rectangular coordinates (x, y, z). For problems involving a sphere, it is appropriate instead to use spherical coordinates (*r*, *θ*, *φ*), with their origin at the centre of the sphere. In this case, the solutions are called spherical harmonics and have azimuthal symmetry (i.e., no *φ*-dependence), with general solutions taking the following form [14,19,23,56,62]:

(16) $$\phi \left(r,\theta \right)=\sum_{l=0}^{\infty }\left(A_{l}r^{l}+\frac{B_{l}}{r^{l+1}}\right)P_{l}\left(\cos\theta \right)$$

where $\;P_{l}(cos\theta )$ are the Legendre polynomials.

At this point, a reminder of some of the fundamentals involved may be of value. Of basic importance is the Fourier analysis of periodic functions, where a function *f*(*t*) of time that is periodic over an interval of time *T* can be synthesized from a sum of weighted sinusoids:

(17) $$f\left(t\right)=\sum_{0}^{T}\left(a_{m}cos\frac{2\pi mt}{T}+b_{m}sin\frac{2\pi nt}{T}\right)$$

Equation (17) is the well-known Fourier series, where *a_m_* and *b_m_* are weighting coefficients determined directly from *f*(*t*) [62,63]. Sine and cosine functions are orthogonal—a property demonstrated by the following integrals:

(18) $$f\left(t\right)=\sum_{m=0}^{\infty }\left(a_{m}cos\frac{2\pi mt}{T}+b_{m}sin\frac{2\pi mt}{T}\right)$$

(19) $$\int_{0}^{T}sin\frac{2\pi mt}{T}sin\frac{2\pi nt}{T}dt=f\left(x\right)=\left\{\begin{array}{c} \frac{T}{2},\;if\;m=n;\;\\ 0,\;if\;m\neq n\;\; \end{array}\right.$$

(20) $$\int_{0}^{T}cos\frac{2\pi mt}{T}cos\frac{2\pi nt}{T}dt=f\left(x\right)=\left\{\begin{array}{c} \frac{T}{2},\;if\;m=n\neq 0;\;\\ 0,\;if\;m\neq n;\;\;\;\;\;\;\;\;\;\;\\ T,\;if\;m=n=0 \end{array}\right.$$

If Equation (17) is multiplied by either $sin\frac{2\pi mt}{T}$ or $cos\frac{2\pi nt}{T}$ and integrated over the time period *T*, all but one of the terms of the infinite summation are zero. Obtaining the best values for *a_m_* and *b_m_* is thus simplified because the infinite series now becomes

(21) $$\begin{array}{cc} a_{n}=\frac{2}{T}\int_{0}^{T}f(t)cos\frac{2\pi nt}{T}dt, & n=1,2,3,., \end{array}$$

(22) $$\begin{array}{cc} b_{n}=\frac{2}{T}\int_{0}^{T}f(t)sin\frac{2\pi nt}{T}dt, & n=1,2,3,., \end{array}$$

(23) $$a_{0}=\frac{2}{T}\int_{0}^{T}f(t)dt.$$

**Figure 3:**
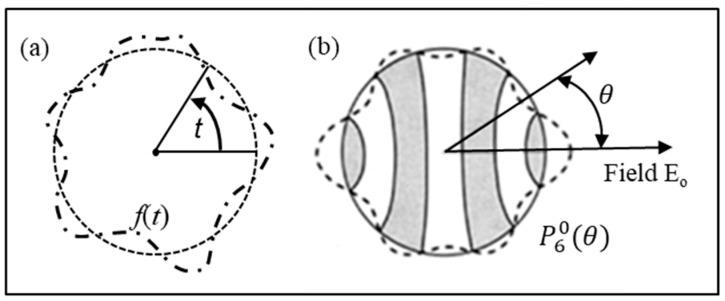
(**a**) A periodic function *f*(*t*), defined by its location around the circumference of a circle, has a fundamental period *T* of 2*π*. (**b**) The shaded and unshaded regions for $P_{6}^{0}(\theta )$ show areas where the function is positive or negative, respectively, when wrapped around a sphere.

This demonstrates that, because sinusoids are orthogonal over the period of *f*(*t*), their use (instead of log functions or exponentials, for example) delivers calculations of *a_m_* and *b_m_* directly from function *f*(*t*). As shown in Figure 3a, formfitting *f*(*t*) around the circumference of a circle, with *t* representing the angle from a fixed radius, gives the fundamental period as *T* = 2*π*. Equation (17) can now be written as

(24) $$f\left(t\right)=\sum_{m=0}^{\infty }\left(a_{m}cos\;mt+b_{m}\sin mt\right)$$

By defining *f*(*t*) in terms of its position around a circle, it can also be represented by a sum of sinusoids around that circle. Spherical harmonic analysis extends this concept to represent a function, defined by position on the surface of a sphere, in terms of latitude (*θ* fixed) and longitude (*φ* fixed). Consider a circle of longitude around the sphere’s surface, with *θ* fixed as shown in Figure 3b; then *f*(*θ*, *φ*) is a function only of longitude (*φ*) and has a fundamental period of 2π. It can be represented by Equation (25):

(25) $$f\left(\theta ,\;\varphi \right)=\sum_{m=0}^{\infty }\left(a_{m}\left(\theta \right)\;cos\;m\varphi +b_{m}\left(\theta \right)\sin m\varphi \right).$$

The same form can be given for any co-latitude (*θ_o_*, *θ*_1_, …, *θ_n_)* on the sphere, with each one having its own set of coefficients *a_m_* and *b_m_*. These coefficients themselves are also functions of co-latitude. The best way to formulate these coefficients is to use *Legendre polynomials*, represented as *P_l_* in Equation (16). $P_{n}^{0}(cos\;\theta )$ is termed the Legendre polynomial of *n*th degree, where for *n* = 0, 1, and 3 [15,19,20,23,56,62],

(26) $$P_{0}^{0}=1;\;P_{1}^{0}=cos\;\theta ;P_{2}^{0}=\frac{3}{2}{cos}^{2}\theta -\frac{1}{2}\;.$$

The function $P_{6}^{0}(\theta )$ is shown along the circumference of a circle in Figure 3b.

### 3.2. Unification Across Laplacian Fields

Figure 1 suggests that the ‘Rosetta Stone’ metaphor may possibly represent unification across Laplacian fields. As defined for engineering and physics applications, fields are physical quantities expressed as a number or tensor, for example, and are functions of both position and time in a region of space. They describe a force or influence that extends through spacetime. A scalar field assigns a single numerical value at a point to describe a quantity with magnitude only—such as temperature, pressure, density, viscosity—while a vector field assigns magnitude and direction to every point to represent a force or flux. Examples include wind speed and direction, a magnetic field created by magnets or moving charges and interacting with other magnets or electric currents, a gravitational field that exerts an attractive force on a mass and is directed towards the centre of the source, and an electric field created by static charges and exerting a force on other charges. The electromagnetic field is an example of a tensor field: it unifies magnetic and electric fields into a single mathematical entity. It may be that the electrome, with multiple components having different physical quantities, is best described as a tensor field. Laplacian fields possess special features. Laplace’s equation describes a field where, *inside* the region of spacetime of interest, there are no sources or sinks for that field. Physically, this corresponds to situations of steady state and equilibrium. Furthermore, following the discussion in Section 3.1, the solutions (i.e., harmonic functions) are seen to have the property that their value at a particular point is the average of values over a surrounding spherical surface. Laplacian fields are thus smooth, minimizing the integral of a potential gradient squared and thus the local field energy. This gives them mathematical and physical stability because perturbations propagate smoothly and globally. Laplace’s equation, above all, governs many apparently different physical systems and so has the potential to represent (or mis-identify) a unifying platform for the ‘Rosetta Stone’ metaphor. The case of Darcy fluid flux—with its velocity field and velocity potential—exemplifies this universal property.

### 3.3. Investigation of a Sphere Placed in a Darcy Flow Field

**Figure 4:**
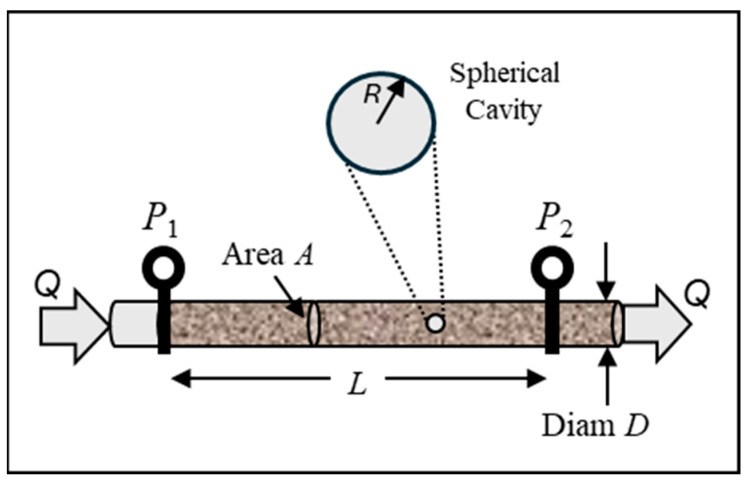
Experimental scheme to determine the perturbations of flow velocity **v** along a tube of diameter *D* packed with porous material caused by a spherical inclusion of porosity *p* and radius *R*. The hydraulic pressures at points *P*_1_ and *P*_2_ remain fixed. The Darcy flux *q* is given by q=Q/A=pv.

The mathematical core of the DEP Clausius–Mossotti factor can be isolated from its specific dielectric context by examining an analogous problem in hydrogeology. Figure 4 depicts a system for investigating the influence of a spherical inclusion on Darcy flux in a circular tube packed with porous material such as sand. Darcy’s Law, formulated by French engineer Henry Darcy in 1856, states that the rate of flow of fluid through a fully saturated porous material is proportional to the pressure difference across it, and inversely proportional to the fluidic resistance of the material [64]. It is a hydraulic version of Ohm’s Law. The proposed experiment in Figure 4 involves hollowing out a small cavity of radius *R*, with *R* << *D*, from the material packed inside the tube. The cavity formed is then refilled with sand or gravel of a different fluidic resistance to the original material. The profile of the fluid flow in the region of the inclusion, when fully saturated, is determined by an array of micro-flow sensors. This procedure is repeated for each type of inclusion material. The author is not aware of attempts to carry out such an experiment, but although it may be of no likely value to hydrogeology, it serves well for our purpose here.

With reference to Figure 4, Darcy’s Law is expressed as

(27) $$Q=-\sigma_{h}A\frac{\left(P_{1}-P_{2}\right)}{L}=-\sigma_{h}A(\nabla P),$$

where *Q* is the measured volumetric flux, $\sigma_{h}$ is the hydraulic conductivity, *A* is the cross-sectional area, and ∇P is the hydraulic gradient. Hydraulic conductivity is thus defined as the rate at which fluid flows through a unit area of a porous material under a unit hydraulic gradient. It depends on the intrinsic coefficient of permeability *k* (units of Darcy), density, and viscosity of the material. Materials with higher porosity have more interconnected voids (pores) for the fluid to flow through and so tend to have higher permeability values, although other factors such as the shape and geometrical connections of the void spaces also participate. The Darcy flux, *q*, is the fluid transported per unit area, so that

(28) q=Q/A

Although the Darcy flux (also known as the Darcy velocity) has units of velocity, it does not represent the actual fluid velocity. In a porous material, the fluid must navigate through the pores that surround the solid particles of the material at a velocity greater than the Darcy velocity. This is the average linear fluid velocity **v** and is related to the Darcy flux and the porosity, *p*, of the material by

(29) q=vp

Porosity is thus a dimensionless number, with values for sand, gravel, and clay-like material across the range from 0.15 to 0.7. This velocity field is the gradient of a scalar function (∇ϕ), namely, the velocity potential *ϕ*. Because differentiation of a scalar function results in a value of zero, the velocity potential is not a uniquely defined parameter. For example, an arbitrary function of time, *f*(*t*), can be added to it without changing its physical nature. Thus, the pumping source ∇P of the potential flow can be modulated (e.g., sinusoidally) in the scheme shown in Figure 4. Potential flow is also characterized by an irrotational velocity field, meaning that the velocity **v** has zero divergence, expressed by

(30) ∇·v=0.

Recognizing that v=−∇ϕ, this equation shows that *ϕ* satisfies the Laplacian equation,

(31) $$\nabla^{2}\phi =0,$$

where $\nabla^{2}\;=\nabla \cdot \nabla$ is the Laplacian operator. Because solutions of Laplace’s equation are harmonic functions, each harmonic function is a solution for potential flow.

### 3.4. Solving Laplace’s Equation for the Darcy Flow Field

The potential functions outside and inside the spherical inclusion, *ϕ_m_* and *ϕ_in_*, respectively, are given by solutions of the following two Laplace equations:

(32) $$\begin{array}{cc} \nabla^{2}\phi_{m}=0; & \;\nabla^{2}\phi_{min}=0 \end{array}$$

The solution of each of these equations is each given by Equation (16). Referring to Figure 4, the potential functions are assumed to be continuous everywhere between *P*1 and *P*2, including at the boundary between the porous medium and the spherical inclusion. Consider first the potential *ϕ_m_* in the medium outside the sphere. At a location far from the influence of the spherical inclusion (i.e., *r* >> *R*), the velocity field will be unchanged from the uniform field v*_m_* in the porous medium before the spherical cavity was carved out. Thus, *ϕ_m_* = −v*_m_* r cosθ, corresponding to the *l* = 1 term in Equation (16), to give

(33) $$\phi_{m}\left(r,\theta \right)=-v_{m}\mathrm{rcos}\theta +\sum_{l=0}^{\infty }\frac{1}{r^{2}}{B_{l}P}_{l}\left(\cos\theta \right)$$

The first term on the right-hand-side of Equation (33) is thus the original, unperturbed velocity field, while the second term involving the infinite summation represents the perturbation due to the spherical inclusion. For our purposes, it is sufficient to consider the Legendre polynomials up to the first degree. From Equation (26), this restricts us to $P_{0}^{0}=1$ and $P_{1}^{0}=cos\;\theta$. Equation (33) thus simplifies to

(34) $$\phi_{m}\left(r,\theta \right)=\left(B_{o}r+\frac{B_{1}}{r^{n+1}}\right)\;P_{n}^{0}\left(\cos\theta \right),$$

which from Equation (33) leads to

(35) $$\phi_{m}\left(r,\theta \right)=-v_{m}r\cos\theta +\frac{B_{1}}{r^{2}}\cos\theta \;\mathrm{for}\;r\geq R$$

*B*_1_ is a constant that can be determined from the boundary conditions. The ‘mathematical physics’ of the perturbation caused by the spherical inclusion can be appreciated by noting that the potential *ϕ*_μ_ of a point dipole of moment, μ, located in a dielectric medium of permittivity, *ε*_o_*ε_m_*, is given by [14,17,19,23]

(36) $$\phi_{\mu }(r,\theta )=\frac{\mu }{4\pi \varepsilon_{o}\varepsilon_{m}}\frac{\cos\theta }{r^{2}}$$

**Figure 5:**
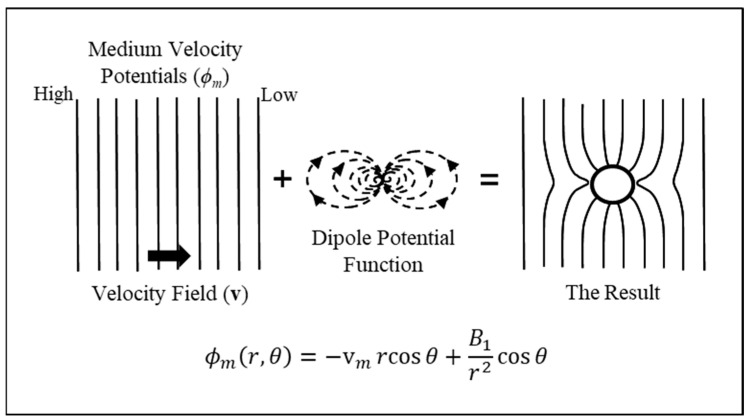
Pictorial representation of Equation (36). The perturbation on the velocity field is caused by a ‘mathematical’ dipole field, located at the centre of the spherical inclusion, being added to it.

The 1/*r*^2^ factor in Equation (35) shows that the perturbation arises from the presence of a ‘mathematical’ dipole field located at the sphere’s centre. A pictorial representation of Equation (35) is given in Figure 5.

To avoid a singularity at *r* = 0 (i.e., 1/*r*^2^ going to infinity), we give the potential function *inside* the spherical inclusion in the following form:

(37) $$\begin{array}{cc} \phi_{in}\left(r,\theta \right)=-Cr\cos\theta & \;\mathrm{for}\;r\leq R \end{array}$$

The boundary conditions must now be determined in order to find *B*_1_ in Equation (35) and *C* in Equation (37). At the surface of the sphere (*r* = *R*), the two boundary conditions below are applied.

Boundary Condition 1

There must be continuity of the tangential component of the velocity field, **v**, across the surface boundary. Since $v_{\theta }=-\frac{1}{r}\frac{\partial \phi }{\partial \theta }$, there must also be continuity of the potential across the boundary:

(38) $$\phi_{in}(R,\theta ){|}_{r=R}=\phi_{m}(R,\theta ){|}_{r=R}$$

Boundary Condition 2

There must be continuity of the normal component of the Darcy flux q=pv across the surface boundary. Since $q=-p\frac{\partial \phi }{\partial \theta }$, the second boundary condition is

(39) $$p_{in}\frac{\partial \phi_{in}}{\partial r}{|}_{r=R}=p_{m}\frac{\partial \phi_{m}}{\partial r}{|}_{r=R}$$

**Figure 6:**
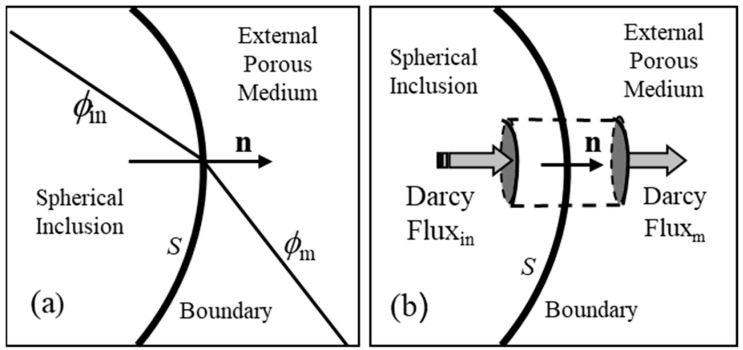
The boundary conditions used to solve Laplace’s equation for Darcy fluid flow. The spherical inclusion and surrounding material have porosity values *p_in_* and *p_m_*, respectively. An infinitely thin surface region *S* bounds the sphere and acts as the boundary with the external material. A vector **n** is drawn normal to *S*. (**a**) The velocity potential functions *ϕ*_in_ and *ϕ*_m_ are continuous across *S*. (**b**) The Darcy flux (**q** = *p***v**) normal to *S* is continuous across *S*.

These two boundary conditions are depicted in Figure 6.

Solving for *B*_1_ and *C* in Equations (35) and (37), using Equations (38) and (39), the result for *ϕ*_m_ is

(40) $$\varphi_{m}=-v_{m}r\cos\theta +\frac{p_{in}-p_{m}}{p_{in}+2p_{m}}R^{3}v_{m}\frac{\cos\theta }{r^{2}}=-v_{m}r\cos\theta +\phi_{1}$$

The component *ϕ*_1_ of the potential outside the spherical inclusion is specifically associated with the dipole quality of the constant *B*_1_, defined by the imposed boundary conditions (ignoring Legendre polynomials beyond the first degree removes the quadrupole, octupole, etc., terms). Comparing this to the potential *ϕ*_μ_ of a point dipole given by Equation (36), the following equality is derived:

(41) $$\mu =4\pi R^{3}\varepsilon_{o}\varepsilon_{m}\left(\frac{p_{in}-p_{m}}{p_{in}+2p_{m}}\right)v_{m}$$

Comparing this to the standard expression for the dipole moment induced in a dielectric sphere when polarized by an external uniform electric field **E**_o_ [12,14],

(42) $$\mu =4\pi R^{3}\varepsilon_{o}\varepsilon_{m}\left(\frac{\varepsilon_{p}-\varepsilon_{m}}{\varepsilon_{p}+2\varepsilon_{m}}\right)E_{o}$$

This equivalence confirms, from the perspective of the surrounding porous medium, that the disturbance caused by the spherical cavity filled with a contrasting material takes a simple dipolar form. It is not a physical dipole, but a mathematical analogue. If a velocity field gradient ∇v was created in the tube shown in Figure 4, would the spherical inclusion experience an additional force equivalent to the DEP force? Of course not. Should we name our newly discovered contrast factor $\left(\frac{p_{in}-p_{m}}{p_{in}+2p_{m}}\right)$ the Clausius–Mossotti factor? Of course not. It has nothing to do with them. Let us simply call it the K-Factor for Darcy flow. The point of this exercise was to show that for a topic widely removed from dielectrics we can obtain a direct mathematical analogy to the classic dielectric sphere problem, with solutions that are formally identical.

Apart from being source free, harmonic, smooth, and boundary-controlled, a Laplacian field is also special because it is universal across physics. To emphasize this, Table 2 contains a compilation of *K*-Factors that can be derived, for the case of spherical particles, by solving Laplace’s equation applicable to other subjects in physics.

**Figure 7:**
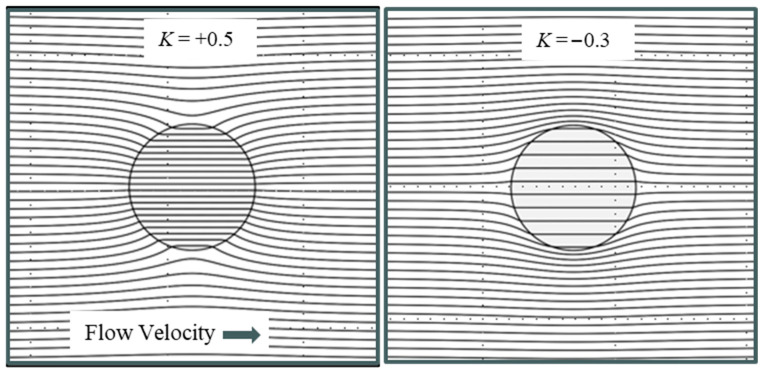
Perturbation of Darcy flow lines for two values of the *K*-factor.

**Figure 8:**
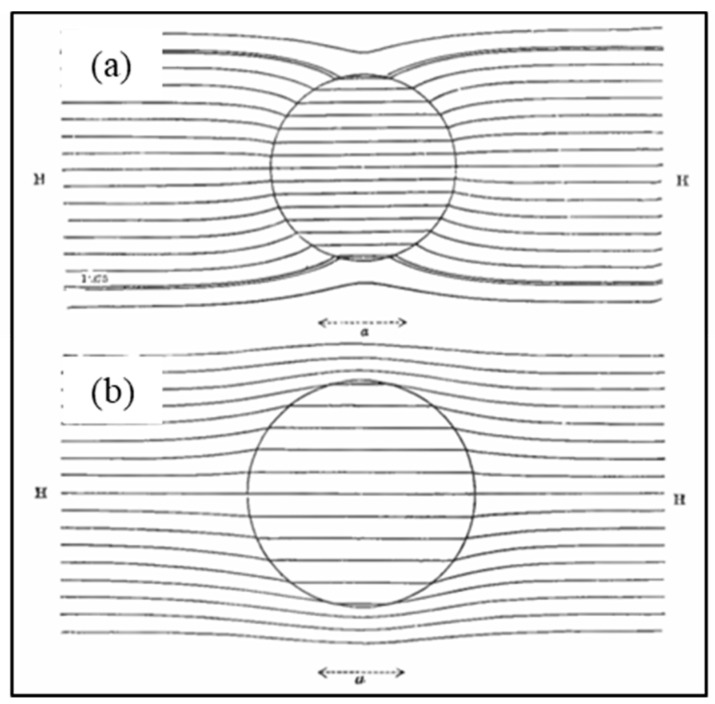
External and internal magnetic field lines for a uniformly magnetized sphere composed of (**a**) paramagnetic material (*μ_r_* = 2.8); (**b**) diamagnetic material (*μ_r_* = −0.48). (obtained from Gray (1898) ([66] Figures 28 and 29, p. 58)).

Depending on the porosity values, our derived contrast factor $K=\left(\frac{p_{in}-p_{m}}{p_{in}+2p_{m}}\right)$ for Darcy flow through sand and clay has values typically in the range +0.55>K>−0.35. Plots of the Darcy flow lines around and through a porous, spherical inclusion are shown in Figure 7 for two values of the *K*-factor. For positive *K* values, the fluid in the surrounding material favours passing through the spherical inclusion at an increased velocity field strength, shown by closer spacing of the flow lines. For negative *K* values, the external fluid is deflected around the spherical inclusion and flows through it at a lower velocity field strength, shown by wider spacing apart of the flow lines. The plots shown in Figure 7 were obtained using GNU Octave-8.2.0 (GUI) software—a free alternative to MATLAB [65]. Similar plots to these can be found for the cases of dielectric flux flow lines for spheres uniformly polarized by an electric field, e.g., [14,19], or, as shown in Figure 8, the external and internal magnetic field lines for a uniformly magnetized sphere.

### 3.5. Questions That Arise

#### 3.5.1. The Moon

Q1: The Moon experiences a non-uniform gravitational field and so the far (dark) side experiences a weaker gravitational attraction than the bright (Earth-facing) side. Does a DEP-like force contribute to the net balance of the centripetal and inertial centrifugal force acting on it?

A1: The short answer is ‘NO’. Mass is always positive, and so, unlike electricity and magnetism (with positive and negative charges, north and south poles), the creation of an induced dipole moment is not possible. The differential (near vs. far side) gravity field produces tidal bulges and internal stresses that cancel pairwise when summed. According to Newtonian physics, the total gravitational force acting on the distributed mass of the Moon equals the product of its total mass and the gravitational field at its centre of mass. Thus, no additional translational force results solely due the gravitational field’s non-uniformity. Energy dissipation associated with the tidal bulges and stresses does change the Moon’s orbit slowly over time (lunar recession).

#### 3.5.2. The Significance of the Factor 2

Q2: The expressions for the molecular CM and DEP CM both contain fractions of following the form:

(43) $$\frac{x-y}{x+2y}$$

The number ‘2’ for the DEP factor arises from the problem of a spherical inclusion in a Laplacian field, solved using spherical harmonics, and specifically the form of the Legendre polynomial, $P_{1}^{0}$ in Equation (33). But why does this number appear in the molecular CM relation?

A2: The appearance of the factor 2 in both cases does not reflect a shared molecular mechanism, but arises from the same mathematical origin: the solution of Laplace’s equation for a spherical region embedded in a uniform field, retaining only the dipolar term. In the DEP problem, this spherical region is the physical particle itself, and the factor 2 arises from enforcing boundary conditions on the electric potential and dielectric displacement flux at the particle surface.

In the molecular theory developed by Clausius, the sphere is instead a conceptual cavity introduced to define the local electric field acting on a molecule. The additional field contribution from polarization charges on the cavity surface is $P/(3\varepsilon_{0})$, a purely geometric result. When this cavity field is combined with the constitutive relation for molecular polarizability, the same algebraic structure—including the factor 2—emerges.

#### 3.5.3. Non-Unique Inverse Problem

Q3: If *K*(ω) can be inferred from DEP measurements on a cell, does this make it a physical observable in the same sense as membrane potential or zeta potential?

A3: No. The Clausius–Mossotti factor is not directly observable; it is a (multi-shell) *model-dependent contrast parameter* inferred by fitting experimental data to a solution of Laplace’s equation under specific assumptions (geometry, linearity, homogeneity, and steady state conditions). While changes in *K*(ω) can correlate with changes in physical properties, its value does not uniquely specify those properties.

Several combinations of parameters (e.g., membrane thickness, capacitance, and conductivity; surface conductance; cytoplasm conductivity and permittivity; and nucleus/cytoplasm volume ratio) can yield a similar *K*(ω). This is a classic non-unique inverse problem. Correlation cannot be equated to identifiability. This does not invalidate DEP, but it does limit what can be inferred without independent constraints.

#### 3.5.4. Implication of Universality of K Across Laplacian Fields

Q4: Given, as shown by Table 2, that identical K-factors arise across electrostatics, hydrodynamics, magnetostatics, and Darcy flow, does this imply a deeper physical unification?

A4: The unification is *mathematical rather than physical*. The shared structure arises because all these systems are governed by Laplace’s equation and involve spherical symmetry with dipolar responses. The resulting contrast factors encode how boundary conditions redistribute a field—not the microscopic mechanisms responsible for that field. In brief, the Clausius–Mossotti factor unifies *how fields respond to contrast*, not *what those fields physically are*.

The exercise in this Section 3 demonstrates that the so-called Clausius–Mossotti factor in DEP is not unique to dielectrics, but is a generic result of *spherical* geometry in a Laplacian field. Hughes [1] approximates the cell to a sphere and assumes that it does not change shape during a DEP experiment. There are pragmatic reasons for doing so—it is easier to determine the diameter of a sphere than the major and minor axes of an ellipsoid, for example. However, the *K*-factors of Table 2 would change *immediately* on shape change during an experiment. Unless the membrane biophysics that controls *V*_m_ and ζ can ‘track’ the cell global geometry in real-time, this could be a fundamental limitation for the Roseta Stone metaphor. The consequences of having a non-spherical particle should therefore be considered.

## 4. Non-Spherical Particles

The purpose here is to draw attention to why a change in cell shape from spherical to an ellipsoid may be off limits for *K*(ω) to act as a direct translator between DEP response and intrinsic electrophysiological state. It may not ‘kill’ the Rosetta Stone metaphor, but could indicate the boundaries for the languages it can translate, and what it cannot. Hughes [1] does draw attention to this potential issue, and so a summary only of the main points, rather than a comprehensive analysis, is merited here.

When spherical symmetry is abandoned, Laplace’s equation remains the governing equation, but the solution is no longer isotropic. There are now direction-dependent polarization factors *L_x_*, *L_y_*, *L_z_*, [14] with

(44) $$L_{x}+L_{y}+L_{z}=1$$

The DEP Clausius–Mossotti Factor $K^{*}(\omega )$ of Equation (1) becomes tensorial. Instead of one scalar contrast factor, there are now three:

(45) $$K_{i}\left(\omega \right)=\frac{\varepsilon_{p}^{*}-\varepsilon_{m}^{*}}{\varepsilon_{m}^{*}+L_{i}{(\varepsilon }_{p}^{*}-\varepsilon_{m}^{*})}$$

where *i* identifies the principal axes (*x*, *y*, *z*). If two of the three axes (*a*, *b*, *c*) of the ellipsoid are equal, it is a spheroid. If *a* = *b* > *c*, the spheroid is oblate; for *a* > *b* = *c*, the spheroid is prolate. For the case of a sphere (*a* = *b* = *c*), then *L_x_* = *L_y_* = *L_z_* = 1/3, and Equation (45) reduces to

(46) $$K_{i}\left(\omega \right)=3\frac{\varepsilon_{p}^{*}-\varepsilon_{m}^{*}}{{\varepsilon_{p}^{*}+2\varepsilon }_{m}^{*}}=3K^{*}\left(\omega \right)$$

**Figure 9:**
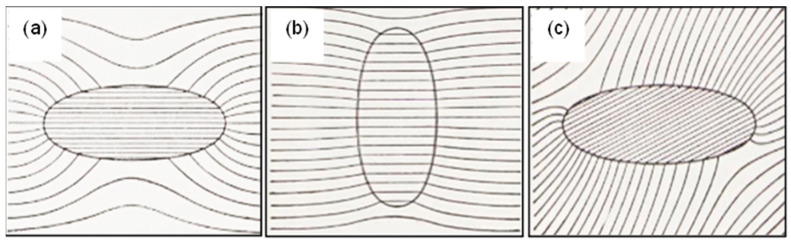
(**a**,**b**): Field applied parallel and perpendicular, respectively, to the major axis and minor axis, respectively, of a prolate spheroid. (**c**): Field applied at 45° to the major axis. A rotational torque is exerted on the spheroid, of magnitude governed by the imaginary component of K*(*ω*).

In the DEP force equation, the number 3 is cancelled in the product with the sphere volume ($\frac{4}{3}\pi R^{3}$). The directional dependence of polarization is demonstrated in Figure 9 for a prolate spheroid.

Importantly, the orientation-dependent DEP response of a non-spherical particle would arise even in the absence of any change in membrane composition, surface charge, or transmembrane potential.

In summary, for non-spherical particles, the DEP Clausius–Mossotti Factor is no longer a scalar, but becomes axis-dependent through geometry-specific polarization factors. This dependence arises purely from the solution of Laplace’s equation requiring the use of ellipsoidal (rather than spherical) surface harmonics and is independent of molecular or physiological processes. For the present, neither the membrane potential nor the zeta potential is known to depend on and encode global cell shape. Both quantities are locally defined: *V_m_* reflects transmembrane ionic gradients and active transport, while *ζ* is determined by surface chemistry and the structure of the electrical double layer. Although cell shape could influence these quantities indirectly through curvature-dependent mechanochemical coupling, for example, this would have to mirror the immediate and tensorial shape-dependence exhibited by $K^{*}(\omega )$. This highlights a fundamental limitation to be considered for the Rosetta Stone metaphor.

## 5. Complex Values for the Universal Contrast Factor K in Table 2

Apart for the case of direct current (DC), the DEP CM factor is complex and frequency-dependent. Complex-valued contrast factors arise in linear systems whenever energy dissipation and storage coexist under time-varying forcing. The parallels (conceptual not mathematical) between Darcy flow and DEP are summarized in Table 3.

For the case of Darcy fluid flow shown in Figure 4, the velocity field is the negative gradient of the velocity potential *ϕ*. As discussed in Section 3.3, the velocity potential is not a uniquely defined parameter because an arbitrary function of time, *f*(*t*), can be added to it without changing its physical nature. Thus, the pumping source ∇P of the potential flow can be modulated, or even applied as an instant step function. If a constant hydraulic pressure is suddenly applied, the Darcy flux will not reach its steady state (static) value immediately, but will approach this gradually with a relaxation time that depends on physical quantities such as the viscosity and density of the fluid and the shape and geometrical connections of the void spaces in the porous material. The Darcy flux *q* is proportional to the velocity field **v**, given by Equation (29), but, because of the phase shift between them, their ratio will depend on frequency. For a periodically modulated driving hydraulic pressure, the resulting velocity field may be written as $v=v_{pk}\cos\omega t$. The Darcy flux will also be periodic in time, but given by

(47) $$q=q_{pk}\cos\left(\omega t-\varphi \right)=q_{1}cos\;\omega t+q_{2}\sin\omega t$$

where *φ* is the phase shift and *q_pk_* is the peak value of the sinusoidal waveform. This introduces two different porosity constants, *p*′ and *p*″, given by $q_{1}=p^{\prime }v_{pk}$ and $q_{2}=p^{\prime \prime }v_{pk}$. The second porosity constant, *p*″, is proportional to irreversible viscous dissipation in the porous network. These relationships can be condensed into two forms by introducing a complex porosity constant and replacing $v=v_{pk}\cos\omega t$ to give the following relationships:

(48) $$p*=p^{\prime }+ip^{\prime \prime };\;v=v_{pk}e^{-i\omega t}$$

where $i=\sqrt{-1}$ and only the real part of $e^{-i\omega t}$ is considered. The choice of sign convention for the exponential is arbitrary and does not affect the physical interpretation. This exercise mirrors that adopted for alternating current dielectric polarization [24] and as well as for Darcy flow can be applied to the other contrast factors listed in Table 2. The complex form of the Clausius–Mossotti factor given by Equation (1), and thus the presence of an imaginary component, therefore reflects a generic phase lag between driving potential and system responses in a dissipative, linear medium, rather than any uniquely electrical or possible biological mechanism. Also, as in other linear inverse problems, the presence of dissipation and storage implies that multiple physical mechanisms may give rise to similar complex response functions.

## 6. Time Constants

The purpose of this section is to clarify the physical origin of time constants that arise as a general result of linear response theory when contrast factors governed by Laplace’s equation are subjected to time-varying forcing. A distinction will be suggested between field-imposed relaxation times and intrinsic biological timescales, and its implications considered for interpreting DEP spectra in terms of the Rosetta Stone metaphor.

In the static limit, contrast factors derived from Laplacian fields are real-valued and time does not explicitly enter the problem. The response of the system is instantaneous and determined solely by boundary conditions and material contrasts. However, once energy storage and energy dissipation coexist, the response to a time-varying forcing necessarily becomes delayed. The system acquires memory of its past states, and its evolution toward equilibrium is no longer instantaneous. This delayed response is mathematically expressed through the emergence of characteristic relaxation times. Such time constants arise whenever a stored quantity (such as electrical charge, elastic strain, or fluid mass) relaxes under dissipative processes (such as electrical conduction, viscous drag, or thermal diffusion). Their existence is therefore not specific to dielectric materials or biological systems, but is a universal feature of linear dissipative systems.

The complex-valued contrast factor (labelled as [CM factor]_DEP_) in Equation (1) ensures that the DEP response is frequency-dependent. This arises from the boundary condition for solving Laplace’s equation involving both a conductive current and a capacitive storage term. As shown in the Appendix A, at the sphere–medium interface these two local terms mix and yield a local resistor-capacitor (RC) type relaxation with a time constant given by the ratio of ε to σ (weighted by the geometry factor 1:2 from spherical matching). The complex electrical conductivity σ*, as defined by Wagner [67] is given as

(49) $$\sigma *=\sigma^{\prime }+i\sigma^{\prime \prime }=\sigma_{c}+i\omega \varepsilon_{o}\varepsilon_{r}$$

where σ_c_ is the steady state (DC) conductivity value. Writing the complex permittivity as

(50) $$\varepsilon^{*}=\varepsilon_{o}\varepsilon_{r}{=\varepsilon_{o}(\varepsilon }^{\prime }-i\varepsilon^{\prime \prime })=\varepsilon_{o}\varepsilon^{\prime }-i\sigma^{\prime }/\omega$$

it is apparent, from Equations (49) and (50), that, at low frequencies, as ω tends to zero, the complex conductivity tends to σ_c_, whilst at high frequencies, as ω tends to infinity, the complex permittivity tends to ε_o_ε′. As shown in the Appendix A, the Maxwell–Wagner interfacial polarization relaxation time $\tau_{MW}$ for a dielectric sphere immersed in a dielectric medium is given by

(51) $$\tau_{MW}=\frac{\varepsilon_{in}+2\varepsilon_{m}}{\sigma_{in}+2\sigma_{m}}$$

It is important to note that this relaxation time for interfacial polarization is fully determined by material contrasts and geometry and exists irrespective of whether the inclusion is biological or inert. DEP is thus inseparable from the existence of a characteristic time constant. It does not arise from any specific molecular mechanism, but is an unavoidable consequence of energy dissipation and energy storage occurring simultaneously in a system subject to time-varying forcing.

The time constant given by Equation (51) is local and confined to the interface of the sphere and surrounding medium, and therefore does not scale with the system’s size. However, this is not the case for Darcy flow.

### Time Constant for Darcy Flow

In this case, we have an incompressible fluid percolating through a rigid porous ‘skeleton’. According to our model for Darcy flow, the evolution with time of the flux is given by

(52) $$\partial_{t}p=\nabla \cdot (D\left(x\right)\nabla p\;\mathrm{with}\;D\left(x\right)=k(x)/\eta$$

This is a diffusion equation with *spatially varying* diffusivity. In contrast to the case for the Maxwell–Wagner time constant (derived in the Appendix A), we have no local storage term of the form $\varepsilon \partial_{t}E$. Instead, the time derivative acts on porosity *p* globally and the driver is the divergence of the flux D∇p. The characteristic time that changes are communicated across distance *L* scales as

(53) $$\tau_{diff}\approx L^{2}/2D$$

So transients can be described as being *domain-scale* diffusive. They are also usually in the form of a sum of exponentials, corresponding to Laplacian eigenmodes [62]. There is no one single local *RC* time constant unless a local storage mechanism is added.

A local storage (capacitive) term could take the form of fluid compressibility (as in storage of fluid in pores) or elastic, porous skeleton compressibility, for example. In such cases, the governing transient equation becomes

(54) $$S(x)\;\partial_{t}p=\nabla \cdot (D\left(x\right)\nabla p),$$

in which *S*(*x*) plays the role of a local storage coefficient (analogous to permittivity *ε*). An ordinary differential equation (ODE) for the dipole term amplitude, of the same *algebraic structure* as Equation (A7), can be derived. A local relaxation time of the form

(55) $$\tau \approx \frac{S_{in}+2S_{m}}{D_{in}+2D_{m}}$$

(or equivalent for the relevant variables) can then be derived based on the procedure given in Appendix A, where it should be noted that geometry factors 1 and 2 remain the same because of the spherical geometry.

In the next section, it is shown that the low-frequency DEP responses of particles that carry a net surface charge are likely to be characterized by a diffusion process with a time constant of the form of Equation (53).

## 7. Membrane Surface Charge

The purpose of this section is to clarify how surface charge enters DEP models, to distinguish between electrokinetic boundary effects and intrinsic cellular regulation, and to assess the implications for the Rosetta Stone metaphor.

**Figure 10:**
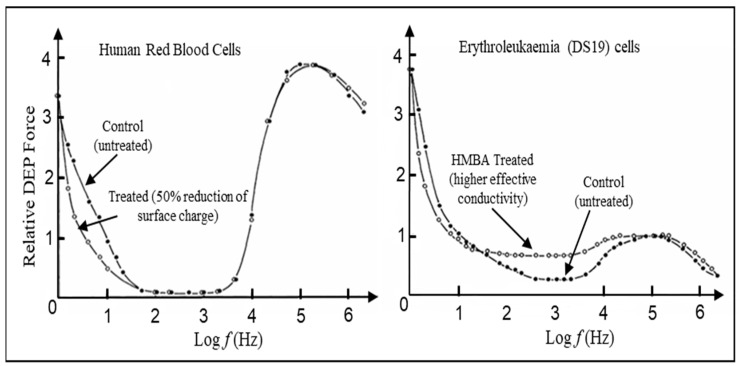
(**Left**): DEP response of human blood cells before and after neuraminidase treatment. (**Right**): DEP response of erythroleukaemia cells before and after treatment with hexamethylene bisacetamide (HMBA). Treatment reduced membrane surface charge and increased effective conductivity [69].

Membrane surface charge, commonly characterized through the zeta potential ζ, plays a distinctive part of DEP. Leaving aside electro-osmosis and fluid convection effects, below ~100 Hz, the DEP response of cells appears to be dominated by membrane surface charge and electrical double layer phenomena. Early investigations of the relationship between DEP, cell-surface charge, and transmembrane potential of mammalian cells [68,69] revealed results such as those presented in Figure 10.

To test the contribution of cell surface charge, red blood cells from fresh human blood were resuspended in a buffered solution containing neuraminidase. This is a well-tested method for reducing charge associated with sialic acid residues on the cell membrane’s surface. Following this treatment, zeta potential values were obtained from the micro-electrophoretic mobility of the blood cells to show that the net cell membrane surface charge had been halved compared to the control cells. The DEP spectra in Figure 10 (obtained using an optical method to detect positive DEP) show that the neuraminidase-treated cells exhibited a clear decrease in positive DEP collection rate below 50 Hz. The unaltered response above 50 Hz also indicated that the effective conductivity and permittivity of the cell membrane was not changed by this treatment, supporting earlier conclusions that cell surface charge influences the DEP behaviour only in the low-frequency range ([69] and the references cited therein). The DS19 clone of Friend murine erythroleukaemic cells progress beyond the colony-forming stage to the stage of terminal differentiation in response to treatment with hexamethylene bisacetamide (HMBA). After this treatment, the average cell diameter decreases by ~12% and the cell surface charge density decreases by 14% [69]. The DEP responses shown in Figure 10 for these cells indicate that, below 10 Hz, the DEP response decreased in line with a reduction in cell surface charge, whilst in the range 20 Hz~30 kHz, a distinct increase in effective conductivity was indicated. The erythroleukaemic cell clones resistant to HMBA treatment did not exhibit changes in DEP behaviour, but on treatment with saponin (to permeabilize the membrane without causing major loss of cytoplasmic protein), a slight increase in cell effective conductivity was observed. The time-dependent decrease in surface charge that accompanied the neoplastic transformation of rat kidney cells was found to mirror the increase in transmembrane potential [68] and was also comparable to the temporal changes in their morphology and virally coded protein content.

Unlike bulk permittivity or conductivity, which enter the contrast factor through volume-averaged material properties, surface charge is inherently interfacial and local. It is therefore tempting to interpret DEP responses that depend on zeta potential as providing direct access to the electrophysiological state of a cell. Surface charge arises from the dissociation of surface groups, adsorption of ions, and specific chemical interactions at the particle–medium interface. In an electrolyte, this charge is screened by a diffuse counter-ion cloud, forming an electrical double layer (EDL) characterized by the Debye length. The zeta potential is not the surface potential itself, but an effective parameter defined at the slipping plane within this double layer. The zeta potential ζ is therefore a *model-dependent* quantity, inferred indirectly from electrokinetic measurements rather than measured directly. As such, ζ reflects the state of the particle–medium interface under specific experimental conditions that specify the hydrodynamic slip plane and the ionic strength of the electrolyte medium, rather than an intrinsic cellular control parameter.

In DEP theory, surface charge does not appear as an independent driving mechanism. Instead, its effects enter through modified boundary conditions at the particle–medium interface, such as those discussed by O’Konski [70], Schwarz [71], and Schurr [72]. Counterions on the surface of a cell carrying surface charge are strongly bound by electrostatic attraction. To escape from the surface into the bulk electrolyte solution, they have to overcome a high potential barrier. Along the membrane surface, however, they can be moved much more easily. If moved tangentially by an external electric field, polarization of the counterion atmosphere induces an additional electric dipole moment of the cell. O’Konski [70] included the field-induced transport of ions on a particle’s surface, together with field-induced bulk flow to and away from the surface of ions from the surrounding electrolyte, as one of the boundary conditions for solving Poisson’s (rather than Laplace’s) equation. Schwarz [71] showed that this displacement of counterions in the double layer is equivalent to the existence of a surface capacitance displaying a diffusion-controlled relaxation. This effect can be expressed by an additional “apparent” dielectric constant of a suspended particle, exceeding its actual dielectric constant at low frequencies by many orders of magnitude. Schurr [72] found fault in Schwarz’s boundary condition that the free charge transported to the surface of the sphere by normal currents may not respond to tangential electric fields. After correcting for this and combining the treatments of O’Konski and Schwarz, Schurr shows that, to account for a tangential surface ionic conductivity, the particle conductivity *σ_p_* is given by

(56) $$\sigma_{p}=\sigma_{bulk}+\sigma_{surface}=\sigma_{bulk}+\frac{2K_{s}}{R}$$

where *R* is the particle radius and *K_s_* the surface conductance. The dielectric properties of the sphere with its counterion layer are equivalent to those of a sphere of uniform conductivity and uniform dielectric constant. Schurr [72] also confirms that the low-frequency dielectric dispersion has a relaxation time given by Equation (53). He even suggests that his correction of Schwarz’s model can be validated by DEP experiments.

Finally, the general solution of Poisson’s equation, given by Lyklema ([73], p. 4.70), for the potential extending beyond the EDL into the bulk medium of a polarized double layer around a sphere is

(57) $$\varphi_{m}^{ff}(r,\theta )=-E_{m}r\cos\theta -f(Du)R^{3}E_{m}\frac{\cos\theta }{r^{2}}$$

In this equation, *f*(*Du*) is a function of the Dukhin number *Du*, which provides a measure of the relative contribution given by *K_s_* in Equation (56). Comparing Equation (57) with the standard solution given by Equation (35) for deriving the CM factor, it is evident that *f*(*Du*) is an analogue of the CM factor for the induced polarization of an electrical double layer. It shares with CM the range of values it can have, namely, 1.0 > *f*(*Du*) > −0.5 [46]. The Dukhin number is defined as the ratio of a particle’s effective surface conductivity to its bulk conductivity:

(58) $$Du\equiv \frac{K_{s}}{R\sigma_{b}}=\frac{\left(K_{s}^{i}+K_{s}^{d}\right)}{R\sigma_{b}}=Du^{i}+Du^{d}$$

The superscripts *i* and *d* in Equation (58) refer to counterion conduction in the Stern (inner Helmholtz) layer and diffuse region of the double layer, respectively. The EDL polarization process can thus be represented through frequency-dependent complex permittivities or conductivities. From this perspective, membrane surface charge alters the effective contrast factor $K\left(\omega \right)$ indirectly by *renormalizing* interfacial transport properties in much the same way that field-imposed relaxation times arise from boundary-controlled dissipation and storage, rather than acting as a separate electrophysiological variable. Surface charge therefore represents another example where the DEP Clausius–Mossotti framework provides a consistent description of field-mediated response, while caution is required in interpreting this response as a direct translation of intrinsic cellular state.

### 7.1. What Does ‘Renormalizing Interfacial Transport Properties’ Imply?

The answer, in brief, is that the presence of surface charge does not introduce a new driving mechanism. Instead, it modifies the *effective values* of the parameters that already appear in the boundary conditions used to solve the Laplace equation and derive K(ω). So the *form* of the Clausius–Mossotti solution stays the same, but the *numbers that go into it change* because the interface behaves differently. For example, the DEP contrast factor depends on the bulk conductivity (*σ_p_*, *σ_m_*) and permittivity (*ε_p_*, ε*_m_*) values, together with the geometry (e.g., the 1:2 weighting). These enter through the boundary conditions of Equations (38) and (39) and Equation (A5) in Appendix A. This is the only place that material properties appear. For the situation where the membrane carries surface charge, then (i) counterions form an electrical double layer, (ii) this layer can conduct ions tangentially along the membrane surface, and (iii) it can store charge capacitively. This introduces additional interfacial transport pathways, specifically surface conductivity *K*_s_ (O’Konski [70]), surface capacitance, and diffusion-controlled polarization (Schwarz [71], Schurr [72]). These processes are local, interfacial, field-driven, and frequency-dependent.

### 7.2. What Becomes ‘Renormalized’ Mathematically?

Surface charge does not *add* a new term to the DEP force equation. Instead, it changes how the interface *appears* to the field. The conductivity is renormalized by employing Equation (56) for the effective conductivity. The *meaning* of *σ_p_* has changed. The permittivity can also be renormalized because the EDL stores charge and relaxes diffusively; it behaves like a surface capacitance. The net effect, as shown by Schwarz [71], is that an apparent permittivity now operates much larger than the molecular permittivity at low frequencies and with its own relaxation time. The contrast factor, K*(ω) of Equation (1) inherits this modification because any change in $\varepsilon_{p}^{*}$ (or $\sigma_{p}^{*}$) automatically changes K*(ω). This is the *only* sense that surface charge affects DEP. These are indirect effects because surface charge does not appear as a new contrast parameter; it does not enter Laplace’s equation as a source term. It does not define a new ‘language’ in the Rosetta Stone sense. Instead, it modifies how ionic current flows along and across the sphere–medium boundary and how charge accumulates and relaxes at this interface. In brief, the same mathematical structure produces a different effective contrast factor.

## 8. Concluding Remarks

This paper examines the Clausius–Mossotti factor as it is used in dielectrophoresis, namely, the DEP CM factor of Equation (1), with particular attention on its proposed role as a unifying ‘Rosetta Stone’ descriptor of electrophysiological cell state [1]. Stepping back from its implied molecular origins and examining its mathematical structure across a broader class of Laplacian field problems, it has been shown here that the characteristic form of the DEP CM factor is not unique to dielectrics or to DEP, but instead arises generically from the solution of Laplace’s equation for spherical and ellipsoidal inclusions subject to appropriate boundary conditions.

From this perspective, the DEP CM factor is best understood as a *universal contrast factor,* encoding how an applied field couples to an object through geometry, contrasting material properties, and interfacial boundary conditions. Its appearance in such subjects as electrostatics, magnetostatics, gravitation, hydrostatics, heat conduction, and Darcy flow reflect the shared mathematical structure of these systems, rather than any deep physical equivalence between them. This universality is a potential strength of the framework for the Rosetta Stone metaphor, but it also imposes intrinsic limits on interpretation. Employing the actual result of some measurements to infer the values of the parameters that characterize the system of interest is a classic inverse problem [74], tackled admirably by Hilton and Hayes [75] in devising a mathematical model to connect DEP measurements and cell properties for bacteria. However, many inverse problems in systems biology may be ill-posed or non-unique in the absence of additional constraints [76,77]. The strong sensitivity of a model output to certain parameters does not imply that those parameters are uniquely identifiable from experimental data. This advocates caution in over-interpreting DEP results such as those shown in Figure 10, for example. Inverse problems in electromagnetics are also well known to deliver non-unique solutions unless strong a priori information is imposed [78]. Identifiability is also not just a property of the experiment alone—it is also a feature of the model structure [79]. This aligns with what is argued here: that the Laplace model itself can limit inference.

With all this in mind, several features commonly invoked to extract biological meaning from DEP spectra were examined here. For ellipsoidal particles the Clausius–Mossotti factor becomes tensorial and unambiguously shape-dependent, reflecting geometry-specific depolarisation factors that arise immediately from the boundary value problem. This dependence has no direct analogue in membrane potential or surface charge, which are locally defined quantities and do not encode global geometry in the same way. Shape changes therefore place a natural boundary on interpreting DEP response as a direct surrogate for intrinsic electrophysiological properties.

The emergence of complex-valued, frequency-dependent contrast factors was shown here to be an unavoidable consequence of energy dissipation and energy storage in linear systems subjected to time-varying forcing. The associated relaxation times are field-imposed, arising from boundary-controlled charge redistribution and interfacial polarization, and exist equally for biological and non-biological particles. While such time constants may correlate with biological processes under specific conditions, they do not in themselves constitute intrinsic biological timescales. Membrane surface charge was similarly shown to influence DEP behaviour through its effect on interfacial boundary conditions, most notably via electrical double-layer polarization and surface conductivity. These effects can be incorporated consistently into the Clausius–Mossotti framework through effective frequency-dependent material parameters. In this sense, surface charge alters the DEP response by renormalizing interfacial transport properties rather than by introducing an independent electrophysiological variable.

Taken together, these considerations suggest a disciplined interpretation of the Clausius–Mossotti factor. The Rosetta Stone metaphor remains heuristically valuable in highlighting the unifying role of linear response and contrast in DEP-based manipulation and characterization. However, the metaphor cannot be literal: the Clausius–Mossotti factor translates *field-mediated response properties*, not intrinsic biological activity and organization. Its power lies in describing how objects interact with applied fields, not in providing a unique or direct decoding of cellular electrophysiology.

Recognizing both the universality and the limitations of the Clausius–Mossotti framework clarifies what information DEP measurements can reliably provide, and where additional constraints or complementary measurements are required. Viewed in this way, the Clausius–Mossotti factor remains a central and powerful concept in dielectrophoresis—not as a universal biological decoder, but as a mathematically grounded bridge between geometry, boundary conditions, and measurable field-mediated response.

## Figures and Tables

**Table 1:** Mossotti provided the qualitative physical picture (corpuscles whose internal charges shift to create dipoles), which Clausius converted into a quantitative microscopic theory by introducing the correct local field, replacing action at a distance with molecular dipoles, and deriving the full polarizability–dielectric relationship.

Feature	Mossotti (1850) [9]	Clausius (1879) [10]
Physical Model	Corpuscles with displaced aether	Molecular Dipoles + Field Theory
Polarization Mechanism	Charge Displacement	Induced and Thermal Dipoles
Local Field	Heuristic	First Rigorous form of the Local Field.
Mathematics	Semi-Quantitative	Quantitative Derivation
Final C-M Relation	Early Form	Modern Clausius–Mossotti Law
Concept	Pre-Maxwellian	Post-Maxwellian Electromagnetism

**Table 2:** A compilation of contrast factors, *K*, that are derived for spherical particles across various topics in physics and engineering. This illustrates the universality of the *K*-factor form across Laplacian fields.

Subject	Potential	Property	K-Factor
Darcy Flux	Velocity *ϕ* $\nabla^{2}\phi =0$	Porosity (a number) *p*	$\frac{p_{p}-p_{m}}{p_{p}+2p_{m}}$
Conductors	Electric *ϕ* $\nabla^{2}\phi =0$	Conductivity (S·m^−1^) *σ*	$\frac{\sigma_{p}-\sigma_{m}}{\sigma_{p}+2\sigma_{m}}$
Magnetostatics	Magnetic *φ_m_* $\nabla^{2}\phi_{m}=0$	Permeability (H·m^−1^) *μ*	$\frac{\mu_{p}-\mu_{m}}{\mu_{p}+2\mu_{m}}$
Geophysics	Gravitational *Φ* $\nabla^{2}\Phi =0$	Density (Kg·m^−3^) *ρ*	$\frac{\rho_{p}-\rho_{m}}{\rho_{p}+2\rho_{m}}$
Hydrodynamics	Velocity *u* ∇·u=0	Viscosity (kg·m^−1^·s^−1^) *η*	$\frac{\eta_{p}-\eta_{m}}{\eta_{p}+2\eta_{m}}$
Heat Conduction	Temperature *T* $\nabla^{2}T=0$	Conductivity (W·m^−1^·K^−1^) *k*	$\frac{k_{p}-k_{m}}{k_{p}+2k_{m}}$

**Table 3:** Conceptual parallels between Darcy flow and DEP are shown, with respect to a complex-valued formulation of the contrast factor *K*(ω).

Feature	Darcy Flow	DEP/Dielectrics
Driving Potential	Hydraulic Pressure *P*	Electrical Potential *ϕ*
Flux	Darcy Flux *q*	Electric Displacement D
Loss	Viscous Dissipation	Joule Heating (σ)
Storage	Compressibility	Capacitance (ε, *C_m_*)
Phase Lag	*q* lags v	P lags E
Imaginary Part	*P*″	ε″

## Data Availability

No new data was created.

## Appendix A

This appendix provides a derivation of the Maxwell–Wagner relaxation time for a spherical inclusion, included here for completeness and to support the discussion in Section 6.

Consider a spherical inclusion (radius R) with permittivity ε_in_ and conductivity σ_in_ embedded in an infinite medium with ε_m_, σ_m_. Apply a time-varying spatially uniform electric field $E_{o}\left(t\right)=-\nabla \phi$ and consider conduction plus displacement current $J=\sigma E+\varepsilon \frac{\partial E}{\partial t}$.

Based on Equation (35), outside the sphere (r > R):

(A1) $$\phi_{m}\left(r,\theta ,t\right)=-E_{o}(t)\mathrm{rcos}\theta +\frac{B(t)}{r^{2}}\cos\theta$$

Based on Equation (37), inside the sphere (r < R):

(A2) $$\phi_{in}\left(r,\theta ,t\right)=-C(t)\mathrm{rcos}\theta$$

The boundary conditions (r = R) to be satisfied are as follows:

1. Continuity of potential:
  (A3) $$-CR=-E_{o}R+\frac{B(t)}{R^{2}}$$
  Thus,
  (A4) $$C\left(t\right)=E_{o}\left(t\right)-\frac{B(t)}{R^{3}}$$
2. Continuity of normal component of total current density, comprising conduction (*σE*) plus displacement current $\left(\varepsilon \partial E/\partial t\right)$ across the sphere–medium interface:
  (A5) $$\begin{array}{c} \left(at\;r=R\right)\\ \left(\sigma_{in}+\varepsilon_{in}\right)\left(-\partial_{r}\phi_{in}\right)=\left(\sigma_{m}+\varepsilon_{m}\right)\left(-\partial_{r}\phi_{m}\right) \end{array}$$

From (A1) and (A2), eliminating *C*(t) from (A4) and cancelling out cos θ terms, (A5) becomes

(A6) $$\left(\sigma_{in}+\varepsilon_{in}\partial_{t}\right)\left(E_{o}-\frac{B}{R^{3}}\right)=\left(\sigma_{m}+\varepsilon_{m}\partial_{t}\right)\left(E_{o}+\frac{2B}{R^{3}}\right)$$

leading to

(A7) $$\left(\sigma_{in}+2\sigma_{m}\right)B+\left(\varepsilon_{in}+2\varepsilon_{m}\right)\frac{\partial B}{\partial t}=R^{3}\left[\left(\sigma_{in}-\sigma_{m}\right)E_{o}+\left(\varepsilon_{in}-\varepsilon_{m}\right)\frac{\partial E_{o}}{\partial t}\right]$$

This is the standard linear ordinary differential equation (ODE) for the (dipole) coefficient B(t), e.g., [62]. It is first-order with constant coefficients.

Consider the special case of a step function or constant applied electric field. If *E*_o_(*t*) = *E*_o_ (constant after *t* = 0) is applied, then $\frac{\partial E_{o}}{\partial t}=0$ and (A7) becomes

(A8) $$\left(\sigma_{in}+2\sigma_{m}\right)B+\left(\varepsilon_{in}+2\varepsilon_{m}\right)\frac{\partial B}{\partial t}=R^{3}\left[\left(\sigma_{in}-\sigma_{m}\right)E_{o}\right]$$

For (say) zero intial B(0), this has the following solution:

(A9) $$B\left(t\right)=B(\infty )\left(1-e^{-t/\tau_{MW}}\right)$$

where the steady amplitude is given by

(A10) $$B\left(\infty \right)=R^{3}\frac{\sigma_{in}-\sigma_{m}}{\sigma_{in}+2\sigma_{m}}E_{o},$$

and the Maxwell–Wagner relaxation time is given by

(A11) $$\tau_{MW}=\frac{\varepsilon_{in}+2\varepsilon_{m}}{\sigma_{in}+2\sigma_{m}}$$

If the field is switched off (e.g., set E_o_(t) = 0 for t > t_0_) then (A7) reduces to the homogeneous ODE:

(A12) $$\left(\varepsilon_{in}+2\varepsilon_{m}\right)\frac{\partial B}{\partial t}=\left(\sigma_{in}+{2\sigma }_{m}\right)B=0$$

In this case, the dipole decays exponentially with the same time constant $\tau_{MW}$, given by (A11).
